# ^31^P magnetization transfer magnetic resonance spectroscopy: Assessing the activation induced change in cerebral ATP metabolic rates at 3 T

**DOI:** 10.1002/mrm.26663

**Published:** 2017-03-16

**Authors:** Chen Chen, Mary C. Stephenson, Andrew Peters, Peter G. Morris, Susan T. Francis, Penny A. Gowland

**Affiliations:** ^1^ Sir Peter Mansfield Imaging Centre University of Nottingham Nottingham Nottinghamshire United Kingdom

**Keywords:** adenosine triphosphate metabolism, magnetization transfer, in vivo ^31^P magnetic resonance spectroscopy, visual stimulation, bioenergetics, brain

## Abstract

**Purpose:**

In vivo ^31^P magnetic resonance spectroscopy (MRS) magnetization transfer (MT) provides a direct measure of neuronal activity at the metabolic level. This work aims to use functional ^31^P MRS‐MT to investigate the change in cerebral adenosine triphosphate (ATP) metabolic rates in healthy adults upon repeated visual stimuli.

**Methods:**

A magnetization saturation transfer sequence with narrowband selective saturation of γ‐ATP was developed for ^31^P MT experiments at 3 T.

**Results:**

Using progressive saturation of γ‐ATP, the intrinsic T_1_ relaxation times of phosphocreatine (PCr) and inorganic phosphate (Pi) at 3 T were measured to be 5.1 ± 0.8 s and 3.0 ± 1.4 s, respectively. Using steady‐state saturation of γ‐ATP, a significant 24% ± 14% and 11% ± 7% increase in the forward creatine kinase (CK) pseudo‐first‐order reaction rate constant, *k*_*1*_, was observed upon visual stimulation in the first and second cycles, respectively, of a paradigm consisting of 10‐minute rest followed by 10‐minute stimulation, with the measured baseline *k*_*1*_ being 0.35 ± 0.04 s^−1^. No significant changes in forward ATP synthase reaction rate, PCr/γ‐ATP, Pi/γ‐ATP, and nicotinamide adenine dinucleotide/γ‐ATP ratios, or intracellular pH were detected upon stimulation.

**Conclusion:**

This work demonstrates the potential of studying cerebral bioenergetics using functional ^31^P MRS‐MT to determine the change in the forward CK reaction rate at 3 T. Magn Reson Med 79:22–30, 2018. © 2017 The Authors Magnetic Resonance in Medicine published by Wiley Periodicals, Inc. on behalf of International Society for Magnetic Resonance in Medicine. This is an open access article under the terms of the Creative Commons Attribution License, which permits use, distribution and reproduction in any medium, provided the original work is properly cited.

## INTRODUCTION

In vivo ^31^P magnetic resonance spectroscopy (MRS) has proven to be a useful technique for the study of bioenergetics associated with different levels of brain activity 1, 2, 3, 4. It not only allows the noninvasive detection of a number of essential phosphate compounds involved in cerebral adenosine triphosphate (ATP) metabolism, which plays a central role in cerebral bioenergetics, but also permits the measurement of cerebral pH and key cerebral ATP metabolic rates and fluxes. The forward ATP synthase (ATPase) reaction rate, measured using ^31^P MRS in resting human and rat brains, has been shown to be consistent with the rate of oxidative phosphorylation 4, 5. A study in rats showed that the forward creatine kinase (CK) and ATPase reaction rates gradually decreased with increasing depth of anesthesia, suggesting a close coupling between forward ATPase reaction rates and the level of brain activity 4. In the human brain, a significant decrease in the ratio of phosphocreatine (PCr) to inorganic phosphate (Pi) and slight increase in pH was observed during ∼13 minutes of photic stimulation 6, whereas a 34% increase in forward CK reaction rate, without significant change in PCr concentration, was reported in response to an 8‐Hz flashing visual stimulation 7. More recently, Barreto et al. 8 reported activation‐induced increases in the Pi/α‐ATP ratio and decreases in the ratio of nicotinamide adenine dinucleotide (NAD) to α‐ATP during both short (1.5 minutes) and long (5 minutes) periods of visual stimulation. No prior studies have assessed the changes in cerebral ATP metabolic rates induced by repeated, prolonged visual stimuli using functional ^31^P MRS.

Cerebral ATP metabolic rates and fluxes can be measured in vivo using ^31^P MRS in combination with magnetization transfer (MT) techniques, such as saturation transfer (ST) 2, 3, 4, inversion transfer 9, 10, and two‐dimensional chemical exchange spectroscopy 11. The ST method is the most commonly used in vivo, due to its high efficiency and methodological simplicity 12. The tightly‐coupled ATPase and CK reactions can be modeled as a three‐pool ^31^P‐spin chemical exchange kinetic network involving ATP, PCr, and Pi 2, 5:

(1) $$\begin{array}{c} \text{PCr} \end{array}+{\text{ADP}}^{-}\;+\;H^{+}\overset{F_{f}^{CK}=k_{1}[PCr]}{\underset{F_{f}^{CK}=k_{-1}[\text{ATP}]}{⇌}}\;\;\begin{array}{c} \text{ATP} \end{array}+\;\;\text{Cr}\;\overset{F_{f}^{\text{ATPase}}=k_{2}[Pi]}{\underset{F_{r}^{\text{ATPase}}=k_{-2}[\text{ATP}]}{⇋}}\begin{array}{c} \text{Pi} \end{array}+\text{ADP}$$

where *k_1_, k_–1_, k_2_, k_–2_* are the forward and reverse reaction rates; $F_{f}^{CK}$, $F_{r}^{CK}$, $F_{f}^{ATPase}$, $F_{r}^{ATPase}$ are the associated fluxes of CK and ATPase reactions; and [ATP], [PCr] and [Pi] are the concentrations of the three phosphate metabolites. Based on this model, the ^31^P MRS‐observable changes in the PCr, ATP, and Pi magnetizations ( $M_{PCr},\;M_{ATP},\;\text{and}\;\;M_{Pi}$) with saturation time (*t*_*sat*_) can be described by the modified Bloch equations 13. For the progressive saturation of γ‐ATP (with boundary condition of $M_{ATP}=0$ at all saturation times), these equations can be simplified to:

(1a) $$M_{PCr}\left(t_{sat}\right)\;=\;M_{PCr}^{0}\left[\left(k_{1}/R_{PCr}^{app}\right)\exp\left(-\;R_{PCr}^{app}t_{sat}\right)+\;1/\left(R_{PCr}^{app}T_{1,PCr}^{int}\right)\right]$$

(1b) $$M_{Pi}\left(t_{sat}\right)\;=\;M_{Pi}^{0}[\left(k_{2}/R_{Pi}^{app}\right)\exp\left(-\;R_{Pi}^{app}t_{sat}\right)+\;1/\left(R_{Pi}^{app}T_{1,Pi}^{int}\right)],$$

where the apparent relaxation rates and longitudinal relaxation times are given by

$$R_{PCr}^{app}=1/T_{1,PCr}^{app}=\left(k_{1}+1/T_{1,PCr}^{int}\right);R_{Pi}^{app}=1/T_{1,Pi}^{app}=\left(k_{2}+1/T_{1,Pi}^{int}\right).$$

$T_{1,PCr}^{int}$, $T_{1,Pi}^{int}$ are the intrinsic longitudinal relaxation times of PCr and Pi, respectively, corresponding to the relaxation rate in the absence of any exchange. In the steady‐state condition, with complete saturation of γ‐ATP, the Bloch equations can be further simplified with the boundary conditions of $\frac{dM_{PCr}}{dt}=0$ and $\frac{dM_{Pi}}{dt}=0$ to give:

(2a) $$k_{1}\;=\left(M_{PCr}^{0}-\;M_{PCr}^{\infty }\right)/\left(M_{PCr}^{\infty }\;T_{1,PCr}^{int}\right)$$

(2b) $$k_{2}=\left(M_{Pi}^{0}-\;M_{Pi}^{\infty }\right)/(M_{Pi}^{\infty }\;T_{1,Pi}^{int}),$$

where $\;M_{PCr}^{\infty }$ and $\;M_{Pi}^{\infty }$ are the steady‐state magnetizations of PCr and Pi. Previous animal and human studies have shown that the intrinsic T_1_s of PCr and Pi are insensitive to changes in physiology 3, 4, 14, 15. To the best of our knowledge, there are no previous reports of the values of the intrinsic T_1_s of PCr and Pi in the human brain at 3 T. Therefore, in this study, $T_{1,PCr}^{int}$ and $T_{1,Pi}^{int}$ values were measured at rest and were then assumed to be unchanged during activation.

The chemical shift between PCr and γ‐ATP is only ∼2.5 ppm, which means that it is difficult to saturate γ‐ATP without partially suppressing the PCr peak 16. In previous studies, BISTRO (B_1_‐insensitive train to obliterate signal) saturation consisting of adiabatic hyperbolic secant pulses with variable amplitudes, has been used for frequency‐selective saturation 2, 17, but combining this with a localization method, such as image‐selected in vivo spectroscopy (ISIS) 18, leads to an excessive specific absorption rate (SAR) 16. More recently, a train of sinc radio frequency (RF) pulses (bandwidth 75 Hz) with constant amplitude has been used to reduce RF power deposition in the MT‐ISIS sequence 16, 19, but this still suffers from the problem of RF bleed‐over. In this study, we use highly selective saturation pulses in the MT‐ISIS sequence, with the aim of effectively suppressing only the γ‐ATP resonance while remaining within SAR constraints.

Conventional in vivo ^31^P spectra from the human brain with no saturation contain a broad, intense phospholipid baseline due to the chemical shift anisotropy of phospholipids, whose resonant frequency depends on their orientation in the field 20, 21. This baseline is significantly reduced by MT saturation at the frequency of γ‐ATP, but this confounds the comparison of ^31^P spectra with and without saturation. To compensate for this, the baseline in the ^31^P spectrum with no MT saturation is normally removed before quantification using a cubic spline or Gaussian fit, or by removing the first few points of data in the time domain 12. However, this procedure can induce extra postprocessing errors or affect signal‐to‐noise ratio (SNR). In this study, we suppressed the phospholipid baseline by applying irradiation in a region of the ^31^P spectrum with no peaks of interest. Because the rotation of the phospholipids is fast compared to the saturation time, phospholipid nuclei that contribute to the broad baseline pass through the orientation corresponding to saturation frequency at some point, and are thus saturated 22.

The principal aim of this study was to measure the changes in ATP metabolic rates in response to repeated, prolonged visual stimulation using ^31^P MRS‐MT. First, the performance of the highly selective saturation sequence for γ‐ATP irradiation and baseline suppression was assessed. Second, longitudinal relaxation times (T_1_) of PCr and Pi at 3 T were determined using the progressive saturation of γ‐ATP. Finally, using the steady‐state saturation of γ‐ATP, the pseudo‐forward rate constants of CK and ATPase reactions (*k*_*1*_ and *k*_*2*_), concentrations of PCr and Pi, and intracellular pH (pH_i_) were quantified during alternating rest and visual stimulation periods.

## METHODS

This study was approved by the medical school ethics committee of the University of Nottingham, and each subject gave informed consent. The study was performed on a 3T Philips Achieva system (Philips Medical Systems, Best, the Netherlands) using a ^31^P transmit–receive 14‐cm diameter loop coil (P‐140). A ^1^H image localizer was acquired at the start of each experiment, and the ^31^P coil was tuned and matched before ^31^P MRS acquisition. Shimming was performed using the Philips pencil beam method, which is based on the FASTMAP method 23.

### Saturation Sequence With High Selectivity

**Figure 1:**
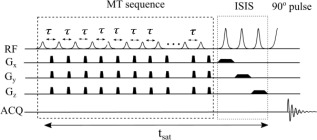
Pulse sequence diagram of the MT sequence in combination with a 3D ISIS localization scheme. The MT sequence comprises multiple amplitude‐modulated RF pulses of constant maximum amplitude and length (2.06 μT, 114.29 ms) interleaved with crusher gradients (4 ms, 10 mT/m) in G_x_, G_y_ and G_z_. The ISIS sequence has three frequency‐modulated HS inversion pulses (5.6 ms, BW = 2.2 kHz). An adiabatic π/2 HS pulse of length 5.4 ms was used for excitation.

Figure 1 shows the MT‐ISIS sequence used in the ^31^P MRS experiments. Prior to the ISIS localization sequence, a highly selective MT sequence was applied comprising multiple amplitude modulated pulses with a hyperbolic secant amplitude envelope (fixed maximum B_1_ of 2.06 μT and pulse duration of 114.29 ms), each having a narrow saturation band (full width at half maximum = 31 Hz; full width at 5% maximum = 69 Hz), interleaved with crusher gradients (4 ms, 10 mT/m) along all three directions (G_x_, G_y_, G_z_). The MT pulse was chosen to provide a saturation profile with a bandwidth that was narrow but sufficient to fully irradiate the γ‐ATP resonance, to have negligible sidebands and to limit energy deposition to allow the use of the pulse train with the ISIS localization sequence. The length of the saturation sequence could be adjusted by varying the number of pulses (*n*_*p*_) and pulse gap (*τ*). The off‐resonance saturation was applied at either −2.5 ppm (relative to the PCr resonance at 0 ppm) to irradiate the γ‐ATP resonance (but also unavoidably the phospholipid baseline) or at 15 ppm to suppress the phospholipid baseline alone.

To assess the performance of this MT‐ISIS sequence, ^31^P spectra with saturation applied at −2.5 ppm (γ‐ATP resonance), + 2.5 ppm, and +15 ppm, respectively, were acquired (N = 1, voxel size = 6 × 6 × 6 cm^3^). Assuming that saturation at ±2.5 ppm leads to the same saturation on the PCr peak, then the comparison of the effect of saturation at +2.5 ppm and +15 ppm of the PCr peak should reveal any direct saturation due to RF bleed‐over (rather than magnetization transfer). Furthermore, comparison of the ^31^P spectra acquired with saturation at −2.5 ppm and +15 ppm should demonstrate if similar suppression of the phospholipid baseline was achieved.

### Progressive Saturation of γ‐ATP: Intrinsic T_1_ Measurement

Six subjects (age range: 22–27 years, four males) were recruited to participate in the γ‐ATP progressive saturation experiment. ^31^P spectra were acquired at resting state from a localized volume (5 × 9 × 7 cm^3^) chosen to cover the occipital lobe using the MT‐ISIS sequence (echo time/repetition time [TR] = 0.1/12,000 ms, samples = 4096, spectral bandwidth = 3000 Hz, number of signal averages (NA) = 24, phase cycles = 8, scan time = 5 minutes). Seven ^31^P spectra were obtained at varying MT saturation times (*t*_*sat*_ = 0, 565, 1058, 2045, 3032, 5005, 8295 ms) using a varying number of saturation pulses (*n*_*p*_ = 0, 3, 6, 12, 18, 30, 50) at a fixed pulse gap, *τ* of 50 ms.

The first scan had no saturation at the γ‐ATP resonance yielding the Boltzmann equilibrium PCr and Pi magnetizations ( $M_{PCr}^{0}$ and $M_{Pi}^{0}$), with the broad baseline attenuated by applying a train of 32 pulses off‐resonance at + 15 ppm. The remaining six γ‐ATP saturation scans were acquired in a random order. The total scan time was approximately 45 minutes. The intrinsic T_1_ of PCr and Pi were determined by fitting $M_{PCr\;}$ and $M_{Pi}$ measured for different values of *t*_*sat*_ in this progressive γ‐ATP saturation experiment, to Eq. [1].

### Functional MRS With Steady‐State Saturation of γ‐ATP: Measurement of Changes in *k*_*1*_ and *k*_*2*_

Nine subjects (age range: 22–27 years, six males) undertook a visual stimulus paradigm which applied two cycles of a 10‐minute rest block followed by a 10‐minute visual stimulation block. The visual stimulation comprised contrast‐defined wedges moving toward or away from a fixation cross of randomized color 24, 25, which was presented through MR‐compatible goggles (VisualSystem; NordicNeuroLab, Bergen, Norway). A median grey background was presented during rest periods. The built‐in diopter correction system and fine‐tuning of pupil distance were adjusted for each subject. Subjects were asked to focus on the fixation point, a small cross, in the center of the field of view. To keep subjects focused during the experiment, they were also asked to press the fiber‐optic response grips (NordicNeuroLab) with their index fingers immediately when the fixation cross turned green.

During each block, two ^31^P spectra (NA = 24) were acquired: one without MT saturation at the γ‐ATP resonance (*t*_*sat*_ = 0 s) to measure Boltzmann equilibrium magnetizations ( $M_{PCr}^{0}$, $M_{Pi}^{0}$), and one with sufficiently long MT saturation time (*t*_*sat*_ = 9820 ms) to measure steady‐state magnetization ( $M_{PCr}^{\infty }$, $M_{pi}^{\infty }$). The total scan time of this session was approximately 50 minutes. The forward rate constants of CK and ATPase reactions (*k*_*1*_ and *k*_*2*_) were calculated based on Eq. [2].

### ^31^P MRS Analysis

The ^31^P data were analyzed in jMRUI version 4 (http://www.jmrui.eu/) 26. The quantification of ^31^P metabolites was performed with the AMARES (Advanced Method for Accurate, Robust and Efficient Spectral fitting) algorithm 27. With soft constraints on signal linewidths and resonant frequencies, the in vivo ^31^P spectra were fitted to 12 individual components for 10 metabolite resonances belonging to phosphoethanolamine, phosphocholine, intracellular Pi, glycerophosphoethanolamine, glycerophosphocholine, membrane phospholipids, PCr, γ‐ATP, α‐ATP, and NAD(H). The doublets of γ‐ATP (−2.5 ppm) and α‐ATP (−7.5 ppm) were both fitted as two peak components with fixed separation of 16 Hz (J‐coupling constant) 28 and with the same linewidth and amplitude. Apodization (line broadening = 15 Hz) was applied to improve the SNR, assisting the visual inspection (in the frequency domain) of the quality of the fit.

Next, the quantified signals (peak areas) of PCr and Pi from the γ‐ATP progressive saturation experiment were least squares fitted to the model in Eq. [1] using MATLAB (The MathWorks, Inc., Natick, MA) to estimate the intrinsic T_1_ of PCr and Pi. The goodness of fit was assessed from the coefficient of determination, *R* ^2^. The forward rate constants of CK and ATPase reactions were calculated from Eq. [2] based on the PCr and Pi signals (*S_PCr,_ S_Pi_*) measured in the steady‐state γ‐ATP saturation experiment. For each block in the visual stimulation paradigm, the concentrations of PCr ([PCr] = *(S*_*PCr*_/*S_γ‐ATP_)* × [ATP]) and Pi ([Pi] = (*S*_*Pi*_/*S_γ‐ATP_)* × [ATP]) were calculated based on the fully relaxed signals from the ^31^P spectra in the absence of γ‐ATP saturation (TR = 12 s), assuming the relatively stable ATP concentration in the human brain to be 3 mM 2, 29. From these, the forward fluxes (in mM/s) were calculated as $F_{f}^{CK}$ = k_1_ × [PCr] and $F_{f}^{ATPase}$ *= k_2_* × [Pi], and converted into units of μmol/g/min, assuming a brain tissue density of 1.1 g/mL 2. The pH_i_ values for each block were determined based on the chemical shift difference ( $\delta_{PCr-Pi}$) between the PCr and intracellular Pi signals 10, 30, 31. Lastly, paired *t*‐tests were performed to test for significant differences in forward reaction rate constants, metabolite concentrations, and pH_i_ between stimulation and rest blocks.

### Error Analysis

To assess the error in the estimate of the intrinsic longitudinal relaxation time ( $T_{1}^{int}$) via the progressive saturation of γ‐ATP, a Monte Carlo (MC) simulation study was performed, based on the time courses of PCr and Pi signals simulated using the model given in Eq. [1], and the assumed $T_{1}^{int}$ s and forward reaction rates ( $T_{1,PCr}^{int}$ = 5 s, $T_{1,Pi}^{int}$ = 3 s, *k*_*1*_ = 0.3 s^‐1^, *k*_*2*_ = 0.2 s^‐1^) [2]. Random normally distributed noise of similar variance as found in the in vivo T_1_ measurements was added to the simulated signal curves, producing 1000 datasets, which were then individually least squares fitted, to predict the average uncertainty (standard deviation) and mean value of the estimated $T_{1}^{int}$ of PCr and Pi.

A second MC simulation was performed to assess the effect of this uncertainty in the estimated $T_{1}^{int}$ values on the resulting measurement of the reaction rate constants (*k*_*1*_ and *k*_*2*_) via the steady‐state saturation of γ‐ATP. For different assumed relative errors in $T_{1}^{int}$ (0%, 20%, 40%), and simulated signals of PCr and Pi (Eq. [1]) at two saturation times (*t*_*sat*_ = 0 and 10 s) with added noise (1000 datasets), *k*_*1*_ and *k*_*2*_ were calculated using Eq. [2], and the coefficients of variation of the estimated values were determined.

## RESULTS

**Figure 2:**
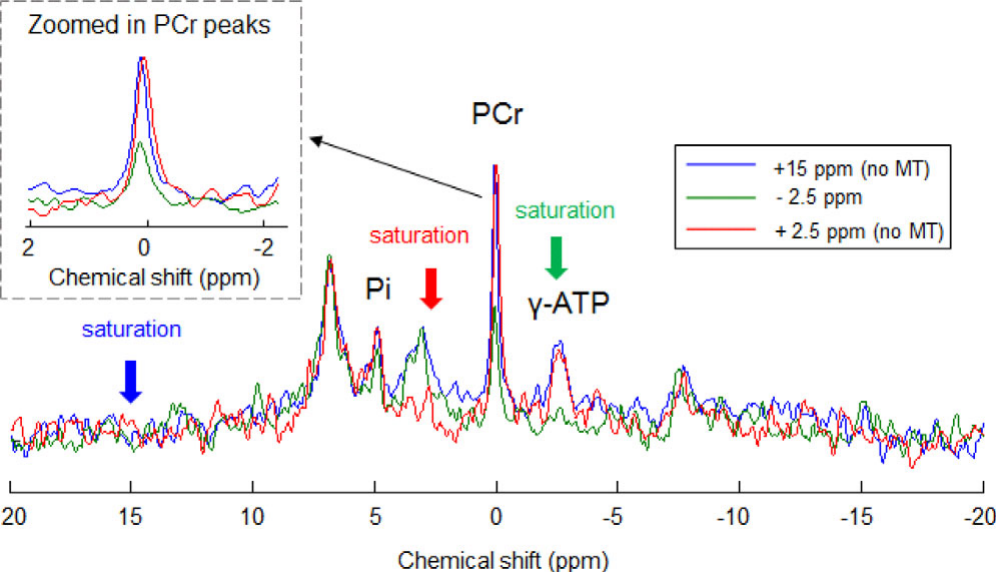
^31^P spectra acquired with saturation at −2.5 ppm and a saturation time (*t*_*sat*_) of 3 s (green), at + 2.5 ppm and a *t*_*sat*_ of 10 s (red), and at + 15 ppm and a *t*_*sat*_ of 5 s as used in the main study (blue). These spectra were acquired from the human occipital region (voxel size = 6 × 6 × 6 cm^3^). They demonstrate that saturation at 2.5 ppm (even for 10 s) has a negligible RF bleed‐over effect on the PCr peak, that off‐resonance saturation of 5s duration provides adequate baseline suppression, and that saturation at −2.5 ppm (for just 3 s) is effective for complete γ‐ATP saturation.

**Figure 3:**
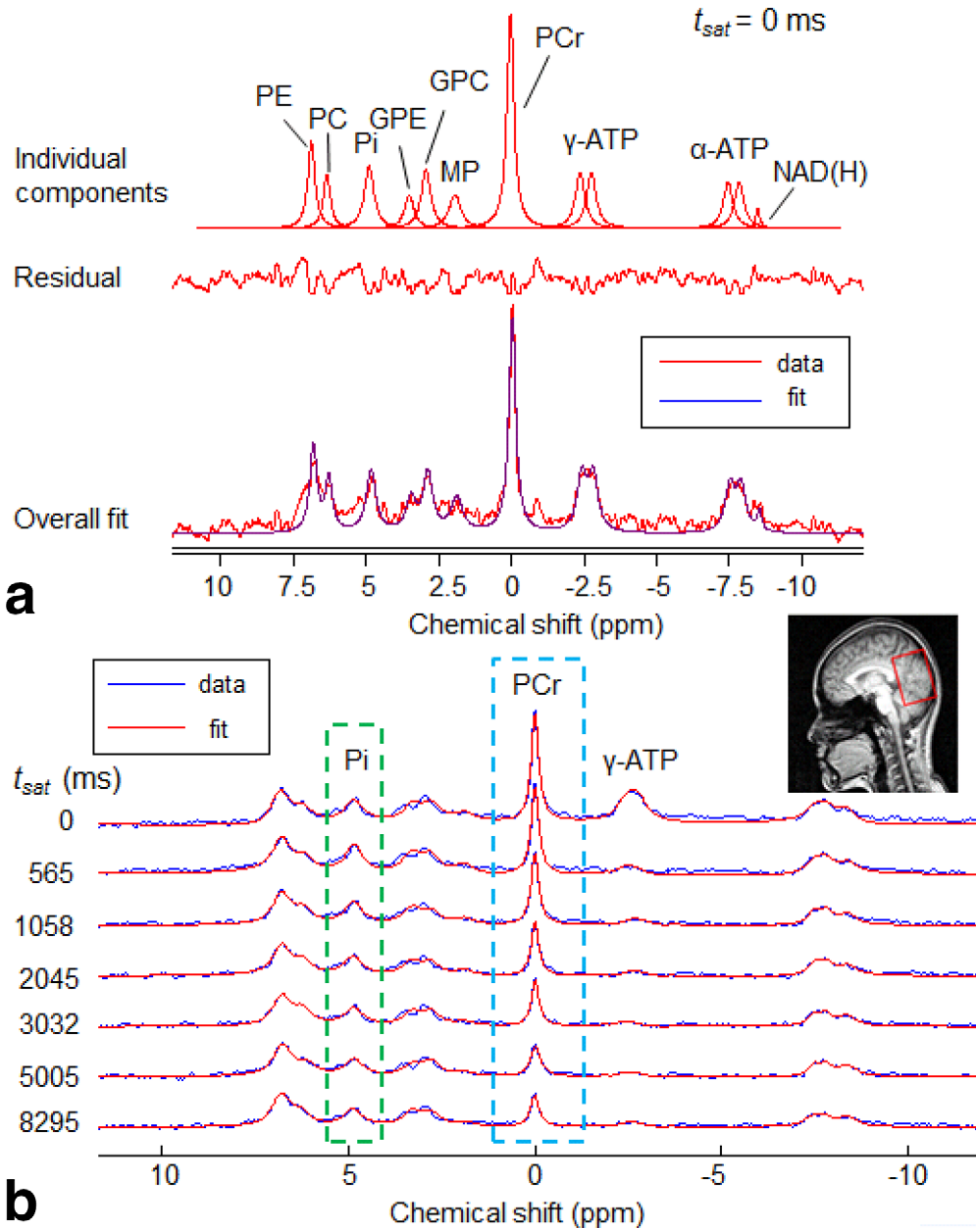
**a**: Plots of 12 individual peak fitting components from 10 metabolites (top row), including phosphoethanolamine (PE), phosphocholine (PC), intracellular inorganic phosphate (Pi), glycerophosphoethanolamine (GPE), glycerophosphocholine (GPC), membrane phospholipids (MP), phosphocreatine (PCr), γ‐adenosine triphosphate (γ‐ATP), α‐adenosine triphosphate (α‐ATP), and nicotinamide adenine dinucleotide (NAD(H)), the residual (middle row), and the ^31^P spectrum acquired (at rest) with no MT saturation at the γ‐ATP resonance and its fitted spectrum in jMRUI for quantification (bottom row). **b**: Plots of the seven ^31^P spectra (voxel size = 5 × 9 × 7 cm^3^) collected with different MT saturation times (*t*_*sat*_ = 0, 565, 1058, 2045, 3032, 5005, 8295 ms) in a representative subject during the progressive saturation of γ‐ATP experiment.

Figure 2 shows that the fully relaxed PCr signals were similar for saturation at +2.5 ppm or + 15 ppm, indicating that the bandwidth of the γ‐ATP saturation profile was sufficiently narrow to prevent RF bleed‐over to the PCr peak. Thus, the suppression of the PCr peak resulting from saturation of γ‐ATP at −2.5 ppm can be considered to be due to saturation transfer only. In addition, it shows that saturation applied at +15 ppm reduced the phospholipid baseline to a similar level to that produced by γ‐ATP saturation at −2.5 ppm. This suggests that more consistent baselines can be achieved between scans with and without γ‐ATP saturation, by applying saturation at +15 ppm (a region without peaks of interest) for the scans acquired without γ‐ATP saturation. Results of spectral peak fitting are shown in Figure 3a for a representative subject. The ^31^P spectra acquired were of excellent spectral quality across all subjects (PCr linewidth, FHWM ∼6–8 Hz, before apodization).

**Figure 4:**
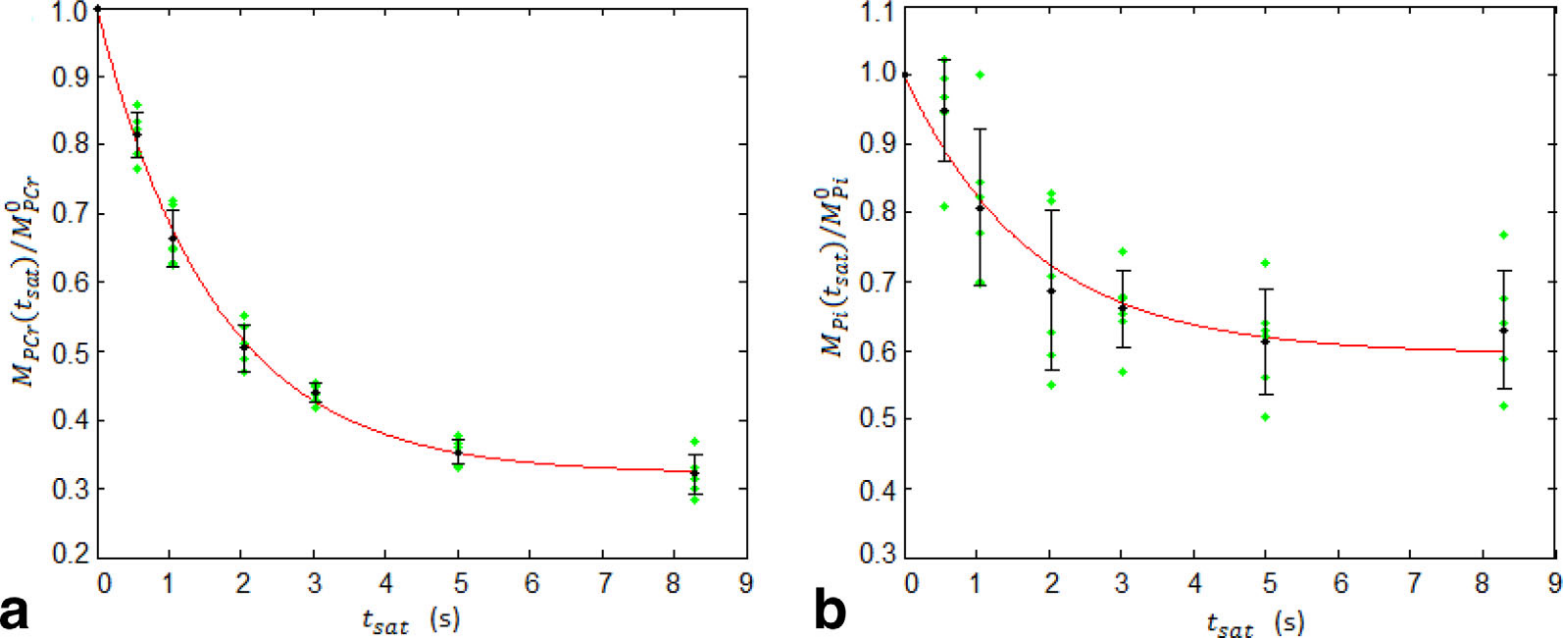
Plots of the normalized magnetization ratio of (a) PCr and (b) Pi as a function of γ‐ATP MT saturation time (*t*_*sat*_ = 0, 565, 1058, 2045, 3032, 5005, 8295 ms), and their least‐square regression curves (red solid line) for the averaged data (N = 6) according to Eqs. (1a) and (1b), respectively. Individual data, group mean, and standard deviation are represented by green dots, black circles, and error bars, respectively. The intrinsic and apparent longitudinal relaxation times of PCr and Pi ( $T_{1,PCr}^{int}$ = 5.1 ± 0.8 s, $T_{1,Pi}^{int}$ = 3.0 ± 1.4 s, $\;T_{1,PCr}^{app}$ = 1.7 ± 0.3 s, $T_{1,Pi}^{app}$ = 1.9 ± 0.7 s), and forward rate constants of the CK and ATPase reactions (*k*_*1*_ = 0.37 ± 0.07 s^−1^, *k*_*2*_ = 0.19 ± 0.07 s^−1^) at rest were determined from these regressions.

Figure 3b illustrates that in the progressive saturation experiment, as expected, the magnitude of PCr signals decreased gradually with increased γ‐ATP saturation time (*t*_*sat*_). The Pi signal decreased more slowly and to a lesser extent with increasing *t*_*sat*_, due to the slower ATPase reaction rate compared to the CK reaction rate [3] and the lower concentration of Pi compared to PCr. Figure 4 shows the normalized PCr and Pi magnetizations (N = 6) plotted against γ‐ATP saturation time, together with the least‐square regression fitting (Eq. [1]) for the group‐averaged data. From this, the intrinsic longitudinal relaxation times ( $T_{1}^{int}$) at 3 T were measured to be 5.1 ± 0.8 s for PCr (*R* ^2^ = 0.99) and 3.0 ± 1.4 s for Pi (*R* ^2^ = 0.7). The uncertainties in the estimated intrinsic T_1_ values were determined from the MC simulation (Table 1), and indicated as expected from Figure 4, that estimated $T_{1,PCr}^{int}$ is more reliable than $T_{1,Pi}^{int}$.

**Table 1:** Assumed and Estimated Intrinsic T_1_ Values of PCr and Pi and the Associated Uncertainties Determined by Monte Carlo Simulation

(s)	True value	Mean	Standard deviation	CV%
$T_{1,PCr}^{int}$	5.1	5.2	0.8	16%
$T_{1,Pi}^{int}$	3	3.5	1.37	40%

**Figure 5:**
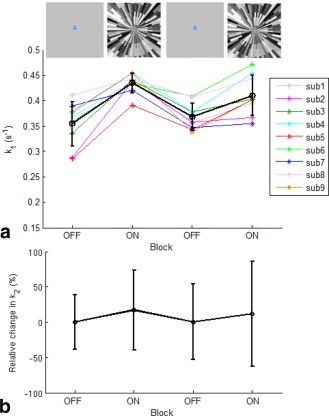
**a**: The forward rate constant of the creatine kinase (CK) reaction (*k*_*1*_) measured within each block during the functional MRS experiments for each subject, and its mean and SD (represented with black circles and error bars). **b**: The mean and SD of the relative change in forward rate constant of the ATP synthase (ATPase) reaction (*k*_*2*_) in each block. Visual stimulation was applied during each ON block.

Using the estimated $T_{1}^{int}$ of PCr, the forward rate constant of the CK reaction (*k*_*1*_) during the functional MRS (fMRS) experiment was determined for each block, and found to be 0.35 ± 0.04 s^−1^ (block1: visual stimulation OFF = baseline), 0.43 ± 0.02 s^−1^ (block2: visual stimulation ON), 0.37 ± 0.03 s^−1^ (block3: visual stimulation OFF), and 0.41 ± 0.04 s^−1^ (block4: visual stimulation ON). Figure 5a shows that the change in the forward rate constant of the CK reaction, *k*_*1*_, between stimulus states was quite consistent across subjects. On average, a 24% ± 14% increase in *k*_*1*_ from baseline was observed during the first visual stimulation ON block (*P* = 2 × 10^−4^). There was no significant change in *k*_*1*_ between the first and second rest (OFF) blocks (*P* = 0.36), indicating that *k*_*1*_ returned to baseline level during the second rest block after visual stimulation. In addition, an 11% ± 7% increase was observed during the second visual stimulation block (*P* = 0.001). There was no significant difference in *k*_*1*_ between the first and second visual stimulation (*P* = 0.08), with *k*_*1*_ elevated by 18% ± 12% when averaged over both visual stimulation periods (*P* = 2 × 10^−6^). The forward exchange fluxes for the CK reaction ( $F_{f}^{CK}$ = k_1_ × [PCr]) were found to increase from 1.32 ± 0.15 mM/s (or 72.2 ± 8.2 μmol/g/min) at rest to 1.56 ± 0.11 mM/s (or 85.5 ± 6.0 μmol/g/min) on visual stimulation (an 18% increase).

Using the estimated $T_{1}^{int}$ of Pi, the forward rate constant of the ATPase reaction (*k*_*2*_) in each simulation block was found to be 0.15 ± 0.05 s^−1^ (stim‐OFF), 0.18 ± 0.09 s^−1^ (stim‐ON), 0.15 ± 0.08 s^−1^ (stim‐OFF), and 0.17 ± 0.10 s^−1^ (stim‐ON). The relative change in *k*_*2*_ showed a similar pattern across blocks to *k*_*1*_ (Fig. 5b), with a trend for an increase in *k*_*2*_ between the first visual stimulation OFF and ON blocks of 21% (*P* = 0.35), and between the second OFF and ON blocks of 12% (*P* = 0.72). Results from the MC simulation (Table 2) show that the coefficient of variation in estimated *k*_*1*_ (CV_k1_% ≈ 7%) is smaller than for *k*_*2*_ (CV_k2_% ≈ 23%), largely independent of the relative errors in the estimated intrinsic T_1_. This indicates that a change less than 7% in *k*_*1*_ or 23% in *k*_*2*_ is unlikely to be detected. The baseline forward exchange flux for the ATPase reaction ( $F_{f}^{ATPase}$ = k_2_ × [Pi]) was determined to be 2.4 ± 0.8 mM/s (or 13.2 ± 4.4 μmol/g/min).

**Table 2:** The Coefficients of Variation in the Estimated Forward Rate Constants of CK and ATPase Reactions (*k_1_, k_2_*) Determined by Monte Carlo Simulation for Different Relative Errors in Intrinsic T_1_s

Assumed relative error in $T_{1}^{int}$	CV% of *k*_*1*_	CV% of *k*_*2*_
0%	6.6%	24.0%
+10%	6.7%	23.3%
+20%	7.3%	22.8%
+40%	6.4%	23.2%

Table 3 shows the PCr/γ‐ATP, Pi/γ‐ATP, NAD(H)/γ‐ATP ratios, and the pH_i_ measured in each block. The concentration of PCr was found to be 3.7 ± 0.3 mM, with no significant difference between rest and visual stimulation (*P* = 0.48), indicating that the activation‐induced change found in $F_{f}^{CK}$ was entirely due to the change in *k*_*1*_. In addition, the concentration of Pi was determined to be 1.6 ± 0.1 mM, with no significant difference between rest and visual stimulation (*P* = 0.99). There was no significant difference observed in the average pH_i_ at rest and on visual stimulation (7.00 ± 0.02, *P* = 0.8). Lastly, the tendency for a decrease in NAD(H)/γ‐ATP ratio upon the first visual stimulation block was not statistically significant (−5%, *P* = 0.31).

**Table 3:** The Signal Ratios of PCr/γ‐ATP, Pi/γ‐ATP, NAD(H)/γ‐ATP, and the Intracellular pH Measured in Each Block of the Visual Stimulation Paradigm

Blocks	PCr/γ‐ATP	Pi/γ‐ATP	NAD(H)/γ‐ATP	pH_i_
[1] Stim‐OFF	1.22 ± 0.09	0.540 ± 0.061	0.157 ± 0.017	6.99 ± 0.04
[2] Stim‐ON	1.25 ± 0.08	0.540 ± 0.052	0.150 ± 0.020	7.01 ± 0.03
[3] Stim‐OFF	1.22 ± 0.07	0.535 ± 0.049	0.157 ± 0.021	7.00 ± 0.03
[4] Stim‐ON	1.23 ± 0.13	0.535 ± 0.069	0.153 ± 0.025	7.00 ± 0.02

## DISCUSSION AND CONCLUSIONS

The magnetization saturation transfer sequence used in this study was designed to provide high frequency‐selectivity for effective saturation of γ‐ATP while keeping the RF power deposition low enough to allow it to be used with ISIS localization with a reasonable TR. This enabled the use of a TR of 12 s (SAR_max_ = 3 W/kg) compared to 30 s or longer required by a conventional BISTRO saturation sequence with ISIS 16. The resulting scan time made it feasible to assess changes in response to repeated visual stimulation in humans. In addition, this study showed that saturation could be applied at +15 ppm to suppress the broad phospholipid baseline in human brain ^31^P spectra, and therefore produce a more consistent baseline across acquisitions with and without MT saturation at the γ‐ATP resonance.

A measurement of $T_{1,\;PCr}^{int}$ (5.1 ± 0.8 s) was obtained at 3 T in this study, which can be compared to 4.9 ± 0.5 s measured in the human brain at 7 T [2] and 3.8 ± 0.6 s in the rat brain at 9.4 T [4]. The decrease in $T_{1,PCr}^{int}$ with the increasing B_0_ field strength suggests that the relaxation of ^31^P spins in PCr is dominated by the chemical shift anisotropy mechanism 32. In contrast, the $T_{1,Pi}^{int}$ measured at 3 T in this study (3.0 ± 1.4 s) was shorter than the values of 3.6 ± 0.7 s, 3.8 ± 0.4 s, and 4.0 ± 0.6 s reported at 4 T, 7 T and 9.4 T 2, 4, 33, respectively, indicating that the relaxation of ^31^P spins in Pi may be dominated by dipolar interactions.

The baseline forward rate constant of the CK reaction (*k*_*1*_) measured using the steady‐state saturation of γ‐ATP (0.35 ± 0.04 s^−1^), is consistent with *k*_*1*_ at resting state obtained from the progressive saturation experiment (0.37 ± 0.07 s^−1^). Early ^31^P studies reported *k*_*1*_ values of 0.3 ± 0.04 s^−1^ and 0.16 ± 0.02 s^−1^ in the human brain regions of predominantly gray matter and white matter, respectively 34, and an average value of 0.42 s^−1^ over the entire human brain 35. Our *k*_*1*_ values are more comparable with the more recently reported values of 0.33 ± 0.04 s^−1^ [2] and 0.32 ± 0.08 s^−1^ 16 measured from the human occipital lobe at rest. In addition, the forward rate constants of ATPase reaction (*k*_*2*_) at rest obtained in this study from the steady‐state and progressive MT experiments (0.15 ± 0.05 s^−1^, 0.19 ± 0.07 s^−1^, respectively) are comparable with each other, and with the previously reported values of 0.17 ± 0.04 s^−1^ [3], 0.18 ± 0.05 s^−1^ [2], and 0.21 ± 0.04 s^−1^ 10.

The cerebral ATP synthesis flux through oxidative phosphorylation estimated from PET measurements of the cerebral metabolic rate of oxygen (CMRO_2_) utilization (CMRO_2_ = 1.71 ± 0.18 μmol/g/min) in the human occipital lobe 36 is 10.3 ± 1.1 μmol/g/min, based on a P:O_2_ ratio of 6 [4]. Our estimate for the forward exchange flux for the ATPase reaction, $F_{f}^{ATPase}$ = 13.2 ± 4.4 μmol/g/min, derived from the ^31^P steady‐state MT measurement of *k*_*2*_ and [Pi] at rest (baseline), is in good agreement with this, as well as values reported in previous ^31^P MT studies (12.1 ± 2.8 μmol/g/min [3] and 8.8 ± 1.9 μmol/g/min [2]), which did not use a localization sequence, but relied on the spatial selectivity of the surface coils used. This suggests that the forward exchange flux of the ATPase reaction measured by the ^31^P MT method reflects the net oxidative ATP phosphorylation rate in the human brain. The possible reasons for this are discussed at length in Lei et al. 3, and the present findings, obtained from a well‐localized volume in the occipital lobe, lend weight to their arguments.

In this study, increases of 24% ± 14% and 11% ± 7% in the pseudo‐forward rate constant of the CK reaction, *k_1_*, were observed in the first and second 10‐minute blocks of visual stimulation, respectively, giving an average increase of 18% ± 12%. This was less than the 34% increase reported previously for a light flashing at 8 Hz [7], and greater than the 8% increase induced by repeated short (20 s) visual stimulation 37. In addition, there was a trend for an increase in the forward exchange rate constant of the ATPase reaction during visual stimulation, although it did not reach statistically significance due to the high coefficient of variation associated with the measurement (Table 2) and the lower SNR. With the benefit of greater SNR, a recent study at 7 T measured a 21% increase in the forward chemical exchange flux of ATPase reaction during repeated short periods of visual stimulation 37.

The baseline PCr/γ‐ATP ratio obtained in this study was 1.22 ± 0.09 (at rest), which is comparable to the previously reported value of 1.3 ± 0.1 measured from the human occipital lobe [2]. In addition, despite the possibility of quantification error due to the limited SNR of the Pi resonance, the baseline concentration of Pi ([Pi] = 1.6 ± 0.1 mM) measured is within the range of previously reported values (0.85–1.7 mM) 3, 10, 30, 38. Some early ^31^P MRS studies in animal 39 and human brains 6 have indicated a decline in PCr concentration and PCr/Pi ratio during visual stimulation. However, no significant difference in the PCr concentration between rest and activation was measured in this study, supporting similar findings reported more recently in the human brain during visual stimulation under normal and mild hypoxic conditions 7, 40, 41 as well as in the rat brain 14. The unchanged PCr concentration and increased pseudo‐forward CK reaction rate (*k*_*1*_) during stimulation can be possibly explained by a slightly elevated ADP concentration 7, 40, which shifts the CK reaction in the direction of ATP synthesis, as well as stimulating glycolysis and lactate production 7, 42. Increased local lactate concentration during functional activation has been found in a few ^1^H MRS studies at 7 T 25, 43, 44, 45. With the same stimulus paradigm, an attenuated response was observed in the second period of 10‐minute visual stimulation for lactate by Lin et al. (25). A similar trend was observed for the forward CK reaction rate in this study. This may relate to neuronal adaptation upon repeated stimulation and requires further investigation.

The pH change during brain functional activation is unclear 46. A slight rise in cerebral pH_i_ was previously reported during photic stimulation 6, 47, whereas a small decrease during checkerboard visual stimulation has also been reported 48. Murashita et al. (41) suggested the change in pH_i_ due to stimulation is age dependent, as the pH_i_ increase found in a middle‐aged group (54.4 ± 10.5 years) was not observed in a younger group (28.9 ± 10.5 years). No significant change in pH_i_ was detected in response to visual stimulation (moving contrast‐defined wedges, 10 minutes) in this study in young healthy volunteers. This agrees with other recent ^31^P human studies 8, 40, in which no change was observed during visual stimulation even in mild hypoxic hypoxia. The limited temporal resolution, sensitivity, and the possible pH heterogeneity in the cortical tissues within the large ^31^P MRS voxel 40, 49 make it difficult to capture possible transient changes in cerebral pH_i_ during the prolonged visual stimulation. Significant decreases of ∼5% and ∼2% in NAD(H)/ATP have been recently reported during short (1.5 minutes) and long (5 minutes) photic stimulations 8. In our study, an insignificant decrease was detected in response to a longer period of visual stimulation (10 minutes).

There are a few possible sources of error leading to potential bias in our results. Firstly, poor or incomplete saturation of γ‐ATP, especially at short saturation time (*t*_*sat*_), would tend to cause an underestimation of *k*_*1*_ and *k*_*2*_ 16 in the progressive saturation experiments. In general, the highly selective saturation used in this study was effective for γ‐ATP irradiation over a large voxel of interest. The residual γ‐ATP signals in the ^31^P spectra acquired at the shortest *t*_*sat*_ (<7% of the unsuppressed γ‐ATP signals) were not much higher than the noise level (∼4%). Secondly, using a short TR could cause partial saturation of the acquired ^31^P signals, especially for scans with long *t*_*sat*_, which would then lead to an underestimation of *k*_*1*_ and *k*_*2*_ in the steady‐state saturation experiments. Based on a model that considers partial saturation [3], the partial saturation effect on $M\left(t_{sat}\right)/M^{0}$ when using a TR of 12 s (as in this study), was found to be small (∼3% for PCr and ∼1% for Pi at the longest *t*_*sat*_ = 10 s). Thirdly, the ^31^P signals from Pi in the intracellular and extracellular compartments were not well resolved at 3 T (this study) or 4 T 33, despite good shimming. Recent studies 50, 51 at ultra–high field have found that the extracellular Pi resonance is insensitive to selective saturation or inversion of γ‐ATP, implying lack of chemical exchange between the extracellular pool of Pi and γ‐ATP. The calculation of *k*_*2*_ based on the total Pi signal may therefore lead to the underestimation of *k*_*2*_. The fact that our measured pH (6.99 ± 0.04) was closer to the previously reported intracellular pH values of 7.06 50 and 7.04 8 than to the extracellular pH value of 7.39 50, indicates that the concentration of Pi and *k*_*2*_ reported in this study are primarily determined by the dominant intracellular Pi signal. In future studies, the design of the ^31^P fMRS experiment could be improved by interleaving the two types of scan (*t*_*sat*_ = 0 and 10 s), as this would enable an increase in the temporal resolution for the *k*_*1*_ and *k*_*2*_ measurements, thus potentially allowing the observation of progressive changes within each stimulation block (assuming that the SNR was sufficient).

This work has demonstrated the effectiveness of a highly selective saturation sequence for selective γ‐ATP irradiation and phospholipid baseline suppression. Our findings suggest that the forward creatine kinase reaction rate is sensitive to increased brain functional activity. Results show the feasibility of using ^31^P MRS‐MT techniques at 3 T for future noninvasive studies of bioenergetics in response to stimulation, and in a variety of neurological and neuropsychiatric disorders.

## References

[1] Chaumeil MM , Valette J , Guillermier M , Brouillet E , Boumezbeur F , Herard AS , Bloch G , Hantraye P , Lenon V . Multimodal neuroimaging provides a highly consistent picture of energy metabolism, validating ^31^P MRS for measuring brain ATP synthesis. Proc Natl Acad Sci U S A 2009;106:3988–3993. 19234118 10.1073/pnas.0806516106 PMC2645913

[2] Du F , Zhu XH , Qiao H , Zhang X , Chen W . Efficient in vivo ^31^P magnetization transfer approach for noninvasively determining multiple kinetic parameters and metabolic fluxes of ATP metabolism in the human brain. Magn Reson Med 2007;57:103–114. 17191226 10.1002/mrm.21107

[3] Lei H , Uğurbil K , Chen W . Measurement of unidirectional Pi to ATP flux in human visual cortex at 7 T by using in vivo ^31^P magnetic resonance spectroscopy. Proc Natl Acad Sci U S A 2003;100:14409–14414. 14612566 10.1073/pnas.2332656100 PMC283605

[4] Du F , Zhu XH , Zhang Y , Friedman M , Zhang N , Uğurbil K , Chen W . Tightly coupled brain activity and cerebral ATP metabolic rate. Proc Natl Acad Sci U S A 2008;105:6409–6414. 18443293 10.1073/pnas.0710766105 PMC2359810

[5] Lei H , Zhu XH , Zhang XL , Uğurbil K , Chen W . In vivo ^31^P magnetic resonance spectroscopy of human brain at 7T: an initial experience. Magn Reson Med 2003;49:199–205. 12541238 10.1002/mrm.10379

[6] Sappey‐Marinier D , Calabrese G , Fein G , Hugg JW , Biggins C , Weiner MW . Effect of photic stimulation on human visual cortex lactate and phosphates using 1H and 31P magnetic resonance spectroscopy. J Cereb Blood Flow Metab 1992;12:584–592. 1618937 10.1038/jcbfm.1992.82

[7] Chen W , Zhu XH , Adriany G , Uğurbil K . Increase of creatine kinase activity in the visual cortex of human brain during visual stimulation: a ^31^P NMR magnetization transfer study. Magn Reson Med 1997;38:551–557. 9324321 10.1002/mrm.1910380408

[8] Barreto FR , Costa TBS , Landim RCG , Castellano G , Salmon CEG . ^31^P‐MRS using visual stimulation protocols with different durations in healthy young adult subjects. Neurochem Res 2014;39:2343–2350. 25227748 10.1007/s11064-014-1433-9

[9] Degani H , Alger JR , Shulman RG , Petroff OA , Prichard JW . 31P magnetization transfer studies of creatine kinase kinetics in living rabbit brain. Magn Reson Med 1987;5:1–12. 3657491 10.1002/mrm.1910050102

[10] Ren J , Sherry AD , Malloy CR . ^31^P‐MRS of healthy human brain: ATP synthesis, metabolite concentrations, pH, and T_1_ relaxation times. NMR Biomed 2015;28:1455–1462. 26404723 10.1002/nbm.3384 PMC4772768

[11] Balaban RS , Kantor HL , Ferretti JA . In vivo flux between phosphocreatine and adenosine triphosphate determined by two‐dimensional phosphorous NMR. J Biol Chem 1983;258:12787–12789. 6630206

[12] de Graaf RA . In Vivo NMR Spectroscopy: Principles and Techniques. Chichester, United Kingdom: John Wiley & Sons; 1998.

[13] Frosén S , Hoffman RA . Study of moderately rapid chemical exchange by means of nuclear magnetic double resonance. J Chem Phys 1963;39:2892–2901.

[14] Sauter A , Rudin M . Determination of creatine kinase kinetic parameters in rat brain by NMR magnetization transfer. Correlation with brain function. J Biol Chem 1993;268:13166–13171. 8514755

[15] Shoubridge EA , Briggs RW , Radda GK . ^31^P NMR saturation transfer measurements of the steady state rates of creatine kinase and ATP synthetase in the rat brain. FEBS Lett 1982;140:289–292. 6282642 10.1016/0014-5793(82)80916-2

[16] Jeong EK , Sung YH , Kim SE , Zuo C , Shi X , Mellon EA , Renshaw PF . Measurement of creatine kinase reaction rate in human brain using magnetization transfer image‐selected in vivo spectroscopy (MT‐ISIS) and a volume ^31^P/^1^H radio frequency coil in clinical 3‐T MRI system. NMR Biomed 2011;24:765–770. 21834000 10.1002/nbm.1636 PMC3143248

[17] de Graaf RA , Luo Y , Garwood M , Nicolay K . B_1_‐insensitive, single shot localization and water suppression. J Magn Reson 1996;113:35–45. 10.1006/jmrb.1996.0152 8888589

[18] Ordidge RJ , Connelly A , Lohman JAB . Image selected in vivo spectroscopy (ISIS): a new technique for spatially selective NMR spectroscopy. J Magn Reson 1986;66:283–294.

[19] Shi X , Sung YH , Kondo DG , Carlson P , Hellem TL , Delmastro KK , Kim S , Zuo C , Jeong E , Renshaw PE . Measurement of creatine‐kinase reaction rate constant in human brain using ^31^P magnetization transfer image selected in‐vivo spectroscopy (MT‐ISIS): a preliminary application to bipolar disorder. In Proceedings of the 19th Annual Meeting of ISMRM, Montréal, Canada, 2011 p. 2541.

[20] Kwee IL , Nakada T . Phospholipid profile of the human brain: 31P NMR spectroscopic study. Magn Reson Med 1988;6:296–299. 3362064 10.1002/mrm.1910060307

[21] McNamara R , Arias‐Mendoza F , Brown TR . Investigation of broad resonances in ^31^P NMR spectra of the human brain in vivo. NMR Biomed 1994;7:237–242. 7848814 10.1002/nbm.1940070507

[22] de Kruijff B , Morris GA , Cullis PR . Application of 31P‐NMR saturation transfer techniques to investigate phospholipid motion and oragnization in model and biological membranes. Biochim Biophys Acta 1980;598:206–211. 7417428 10.1016/0005-2736(80)90281-3

[23] Gruetter R . Automatic, localized in vivo adjustment of all first‐ and second‐order shim coils. Magn Reson Med 1993;29:804–811. 8350724 10.1002/mrm.1910290613

[24] Wandell BA , Brewer AA , Dougherty RF . Visual field map clusters in human cortex. Philos Trans R Soc Lond B Bio Sci 2005;360:693–707. 15937008 10.1098/rstb.2005.1628 PMC1569486

[25] Lin Y , Stephenson MC , Xin L , Napolitano A , Morris PG . Investigating the metabolic changes due to visual stimulation using functional proton magnetic resonance spectroscopy at 7T. J Cereb Blood Flow Metab 2012;32:1484–1495. 22434070 10.1038/jcbfm.2012.33 PMC3421086

[26] Naressi A , Couturier C , Devos JM , Janssen M , Mangeat C , de Beer R , Graveron‐Demilly D . Java‐based graphical user interface for the MRUI quantitation package. MAGMA 2001;12:141–152. 11390270 10.1007/BF02668096

[27] Vanhamme L , van den Boogaart A , Van Huffel S . Improved method for accurate and efficient quantification of MRS data with use of prior knowledge. J Magn Reson 1997;129:35–43. 9405214 10.1006/jmre.1997.1244

[28] Jung WI , Staubert A , Widmaier S , Hoess T , Bunse M , van Erckelens F , Dietze G , Lutz O . Phosphorus J‐coupling constants of ATP in human brain. Magn Reson Med 1997;37:802–804. 9126956 10.1002/mrm.1910370525

[29] Hetherington HP , Spencer DD , Vaughan JT , Pan JW . Quantitative 31P spectroscopic imaging of human brain at 4 Tesla: Assessment of gray and white matter differences of phosphocreatine and ATP. Magn Reson Med 2001;45:46–52. 11146485 10.1002/1522-2594(200101)45:1<46::aid-mrm1008>3.0.co;2-n

[30] Barker PB , Butterworth EJ , Boska MD , Nelson J , Welch KM . Magnesium and pH imaging of the human brain at 3.0 Tesla. Magn Reson Med 1999;41:400–406. 10080290 10.1002/(sici)1522-2594(199902)41:2<400::aid-mrm26>3.0.co;2-e

[31] Petroff OAC , Prichard JW , Behar KL , Alger JR , den Hollander JA , Shulman RG . Cerebral intracellular pH by ^31^P nuclear magnetic resonance spectroscopy. Neurology 1985;35:781–788. 4000479 10.1212/wnl.35.6.781

[32] Evelhoch JL , Ewy CS , Siegfried BA , Ackerman JJ , Rice DW , Briggs RW . 31P spin‐lattice relaxation times and resonance linewidths of rat tissue in vivo: dependence upon the static magnetic field strength. Magn Reson Med 1985;2:410–417. 4094555 10.1002/mrm.1910020409

[33] Du F , Cooper A , Lukas SE , Cohen BM , Öngür D . Creatine kinase and ATP synthase reaction rates in human frontal lobe measured by ^31^P magnetization transfer spectroscopy at 4T. Magn Reson Imaging 2013;31:102–108. 22898695 10.1016/j.mri.2012.06.018 PMC3501567

[34] Cadoux‐Hudson T , Blackledge M , Radda G . Imaging of human brain creatine kinase activity in vivo. FASEB J 1989;3:2660–2666. 2629743 10.1096/fasebj.3.14.2629743

[35] Bottomley PA , Hardy CJ . Mapping creatine kinase reaction rates in human brain and heart at 4 tesla saturation transfer ^31^P NMR. J Magn Reson 1992;99:443–448.

[36] Fox PT , Raichle ME , Mintun MA , Dence C . Nonoxidative glucose consumption during focal physiological neural activity. Science 1988;241:462–464. 3260686 10.1126/science.3260686

[37] Lee BY , Zhu XH , Chen W . Elevated ATP synthase and creatine kinase activities in human visual cortex during visual stimulation: a ^31^P NMR magnetization transfer study at 7T. In Proceeding of the 22nd Annual Meeting of ISMRM, Milan, Italy, 2014 p. 12.

[38] Erecińska M , Silver IA . ATP and brain function. J Cereb Blood Flow Metab 1989;9:2–19. 2642915 10.1038/jcbfm.1989.2

[39] Mora B , Narasimhan PT , Ross BD , Allman J , Barker PB . ^31^P saturation transfer and phosphocreatine imaging in the monkey brain. Proc Natl Acad Sci U S A 1991;88:8372–8376. 1924297 10.1073/pnas.88.19.8372 PMC52510

[40] Vidyasagar R , Kauppinen RA . 31P magnetic resonance spectroscopy study of the human visual cortex during stimulation in mild hypoxic hypoxia. Exp Brain Res 2008;187:229–235. 18259737 10.1007/s00221-008-1298-8

[41] Murashita J , Kato T , Shioiri T , Inubushi T , Kato N . Age‐dependent alteration of metabolic response to photic stimulation in the human brain measured by ^31^P MR‐spectroscopy. Brain Res 1999;818:72–76. 9914439 10.1016/s0006-8993(98)01285-2

[42] Hemmer W , Wallimann T . Functional aspects of creatine kinase in brain. Dev Neurosci 1993;15:249–260. 7805577 10.1159/000111342

[43] Schaller B , Xin L , O’Brien K , Magill AW , Gruetter R . Are glutamate and lactate increases ubiquitous to physiological activation? A ^1^H functional MR spectroscopy study during motor activation in human brain at 7 Tesla. Neuroimage 2014;93:138–145. 24555953 10.1016/j.neuroimage.2014.02.016

[44] Bednařík P , Tkáč I , Giove F , DiNuzzo M , Deelchand DK , Emir UE , Eberly LE , Mangia S . Neurochemical and BOLD responses during neuronal activation measured in the human visual cortex at 7 Tesla. J Cereb Blood Flow Metab 2015;35:601–610. 25564236 10.1038/jcbfm.2014.233 PMC4420878

[45] Mangia S , Tkáč I , Logothetis NK , Gruetter R , Van de Moortele PF , Uğurbil K . Dynamics of lactate concentration and blood oxygen level‐dependent effect in the human visual cortex during repeated identical stimuli. J Neurosci Res 2007;85:3340–3346. 17526022 10.1002/jnr.21371

[46] Kauppinen RA , Williams SR . Use of NMR spectroscopy in monitoring cerebral pH and metabolism during systemic and focal Acid‐Base Disturbances In: Kaila K, Ransom BR, eds. pH and Brain Function. New York, NY: Wiley‐Liss; 1988: 605–619.

[47] Rango M , Bozzali M , Prelle A , Scarlato G , Bresolin N . Brain activation in normal subjects and in patients affected by mitochondrial disease without clinical central nervous system involvement: a phosphorus magnetic resonance spectroscopy study. J Cereb Blood Flow Metab 2001;21:85–91. 11149672 10.1097/00004647-200101000-00011

[48] Magnotta VA , Heo H‐Y , Dlouhy BJ , Dahdaleh NS , Follmer RL , Thedens DR , Welsh MJ , Wemmie JA . Detecting activity‐evoked pH changes in human brain. Proc Natl Acad Sci U S A 2012;109:8270–8273. 22566645 10.1073/pnas.1205902109 PMC3361452

[49] Pirttilä TR , Kauppinen RA . Regulation of intracellular pH in guinea pig cerebral cortex ex vivo studied by 31P and 1H nuclear magnetic resonance spectroscopy—role of extracellular bicarbonate and chloride. J Neurochem 1994;62:656–664. 8294928 10.1046/j.1471-4159.1994.62020656.x

[50] Tiret B , Brouillet E , Valette J . Evidence for a “metabolically inactive” inorganic phosphate pool in adenosine triphosphate synthase reaction using localized ^31^P saturation transfer magnetic resonance spectroscopy in the rat brain at 11.7 T. J Cereb Blood Flow Metab 2016;36:1513–1518. 27354096 10.1177/0271678X16657095 PMC5012527

[51] Ren J , Sherry AD , Malloy CR . Amplification of the effects of magnetization exchange by ^31^P band inversion for measuring adenosine triphosphate synthesis rates in human skeletal muscle. Magn Reson Med 2015;74:1505–1514. 25469992 10.1002/mrm.25514 PMC4792267

